# Genetic Mapping of Quantitative Trait Loci Contributing to Variation in In Vitro Deoxynivalenol Levels in *Fusarium graminearum*

**DOI:** 10.3390/toxins18080322

**Published:** 2026-07-25

**Authors:** Upasana Dhakal, Christopher Toomajian

**Affiliations:** Department of Plant Pathology, Kansas State University, Manhattan, KS 66506, USA; dhakalu@oregonstate.edu

**Keywords:** *Fusarium graminearum*, genome-wide association studies, trichothecene, deoxynivalenol, 15-acetyl-deoxynivalenol

## Abstract

Fusarium head blight (FHB) caused by *Fusarium graminearum* is a major disease of wheat and barley worldwide. Besides causing yield loss, *F. graminearum* also contaminates infected grains with trichothecene mycotoxins such as deoxynivalenol (DON) and its acetylated derivatives. Field isolates of *F. graminearum* vary in the amount of mycotoxins produced, both on infected wheat heads and in controlled laboratory experiments. Genes encoding the enzymes responsible for trichothecene mycotoxin biosynthesis are already characterized, but additional genes responsible for the variation in amounts of mycotoxins detected within and among populations remain to be identified. We measured levels of trichothecenes produced in vitro in a sample of 151 *F. graminearum* field isolates. Genome-wide association performed with these measurements identified 10 quantitative trait loci (QTL) associated with variation in DON and/or 15ADON levels. The candidate regions contain many functionally characterized genes, including the Swr1p helicase gene, an MFS transporter, and multiple other transmembrane transporters that may relate to the fungus’ ability to transport trichothecenes across membranes for sequestration or export. These results help to characterize the genetic factors that influence variability in trichothecene levels, which contribute to our understanding of trichothecene levels on infected grain and may lead to strategies to mitigate this toxin contamination.

## 1. Introduction

Fusarium head blight (FHB) is a major disease of wheat and barley worldwide. Several *Fusarium* species can cause FHB, but in North America the disease is primarily caused by *Fusarium graminearum.* The economic loss due to FHB on wheat in 29 U.S. states and Ontario, Canada exceeded $100 million annually from 2018 to 2021 [1]. The impact of FHB is twofold. First, infection results in poor grain filling, leading to direct yield reduction [2]. Second, *F. graminearum* can contaminate infected grains with trichothecene mycotoxins such as deoxynivalenol (DON), 3-acetyl-deoxynivalenol (3ADON), 15-acetyl-deoxynivalenol (15ADON), nivalenol (NIV), and NX-2 [3]. DON binds to ribosomes, inhibits protein synthesis, and can cause acute health problems in animals, such as vomiting, reduced weight gain and other chronic health issues [4]. DON and its related mycotoxins significantly reduce the marketability of grain. Farmers often face steep price discounts, if they can sell the grain at all, when DON concentrations exceed the legal threshold.

Enzymes required for trichothecene biosynthesis are encoded by 15 *TRI* genes. Among them, 12 of these genes are grouped together in a single gene cluster termed the *TRI* cluster. Two other loci beside the *TRI* cluster are known. Two genes, *TRI1* and *TRI16,* are located together in the *TRI1-TRI16* cluster, while *TRI101* is not located close to any other genes with a known role in trichothecene biosynthesis [5,6]. The *TRI* cluster includes the *TRI5* gene, trichodiene synthase, that is responsible for the first step in trichothecene biosynthesis [7]. In the absence of *TRI5*, *F. graminearum* can infect the wheat spike but the disease does not progress much beyond the point of infection, demonstrating the important role in virulence that DON plays in this fungus [8]. The *TRI* cluster also includes the transcription factors *TRI6* and *TRI10*, which are major positive regulators of the trichothecene biosynthesis genes. The biosynthetic pathway includes other important enzymes such as cytochrome P450 monooxygenases, a hydroxylase, and acetyltransferases.

Allelic variation in *TRI* genes is associated with variation in trichothecene biosynthesis in *F. graminearum* isolates. Allelic variation in the *TRI1* locus determines if an isolate produces DON or NX-2. In DON producers, both C-7 and C-8 positions are hydroxylated while only C-8 is hydroxylated in NX-2 producers [9]. Similarly, differential deacetylation activity of *TRI8* is associated with acetylation of the C-3 and C-15 positions in 3ADON and 15ADON producers, respectively [6]. Likewise, *TRI7* and *TRI13* are non-functional in DON producing isolates but functional in NIV producers, where *TRI13* is responsible for C-4 hydroxylation [6].

In *F. graminearum*, trichothecenes are synthesized in toxisomes, which are small organelles induced during trichothecene induction and made of remodeled endoplasmic reticulum arranged as organized smooth endoplasmic reticulum [9]. After synthesis, trichothecenes are exported out of toxisomes. The *TRI12* gene codes for a major facilitator transporter and works as an efflux pump to export trichothecenes [10]. It is hypothesized that other transporters along with *TRI12* play a role in the export and compartmentalization of trichothecene, but the specific transporters and their roles are yet to be identified.

The *TRI* genes are the best-characterized genes related to trichothecene biosynthesis. However, it is highly likely that there are other genes, yet to be identified, with major roles in regulating trichothecene synthesis and excretion. In this study, we used genome-wide association (GWAS) to identify novel genomic loci associated with population variation in the amount of DON and 15ADON detected under uniform experimental conditions.

## 2. Results

### 2.1. Sample Composition, Population Assignment, and Linkage Disequilibrium Decay

Our investigation of variation in mycotoxin levels used a diverse set of 151 isolates chosen from a larger set of *F. graminearum* isolates characterized in our previous population genomics study [11]. This previous study describes single nucleotide polymorphism (SNP) calling from genotyping by sequencing (GBS) reads and the inference of *TRI* loci genotypes and population membership for each isolate used in this study [11]. Most isolates (147 of 151) were assigned to the previously defined North America 1 (NA1) population [12], and an additional four were not assigned to a single population, due to evidence that their ancestry is derived from multiple characterized populations. Of the 151 isolates, 150 have *TRI* genotypes consistent with the 15ADON chemotype and one is 3ADON. Most isolates were collected from Montana, Minnesota, North Dakota, and New York, with single isolates collected from Louisiana and Virginia and the PH-1 isolate from Michigan [13]. Isolates other than PH-1 were collected between 1999 and 2013. Additional information is available (Table S1) on the isolates used in this study, including the SRA accession numbers for the nucleotide sequence data from each.

Patterns of linkage disequilibrium (LD) decay capture the association between genetic variants near each other on the same chromosome. These patterns help to define the windows around statistically associated genetic markers in which causative genetic variants may lie, especially in cases when only a portion of the variants across the genomes of a sample are genotyped and available for association testing, as is the case with our GBS data [14]. The documented LD patterns for existing populations can help describe LD patterns within our study sample. On average, LD decreases to half of its highest value within 3 kb for the NA1 population [11]. The isolates examined in the current study are essentially a sample from the NA1 population, so we expect the same LD decrease to occur within 3 kb of either side of the causative variant. Other genetic variants segregating in our sample and not captured by our GBS, and thus not included in our GWAS, but within 3 kb of associated markers, may be the true causes of some of the trait variation. As patterns of LD can vary across the genome, in some cases the true causal variants may be greater than 3 kb away from the significantly associated marker.

### 2.2. Distribution of Phenotypic Measurements

All 151 isolates were grown in vitro to assess the levels of four trichothecene mycotoxins: DON, 15ADON, 3ADON and NIV. All but three isolates (23310, 24309, 23511) produced levels of DON and 15ADON above the limit of quantitation (LOQ) in one or more of the three replicates, and 135 isolates produced amounts above the LOQ across all three replicates. Neither NIV nor 3ADON were included in the GWAS analysis. NIV did not exceed the LOQ for any isolate. 3ADON exceeded the LOQ across all three replications for 27 isolates, with nearly half of the isolate × replicate measurements not exceeding the LOQ. As all but one isolate had the 15ADON *TRI* genotype, we expected to see little to no 3ADON produced by most isolates.

**Figure 1:**
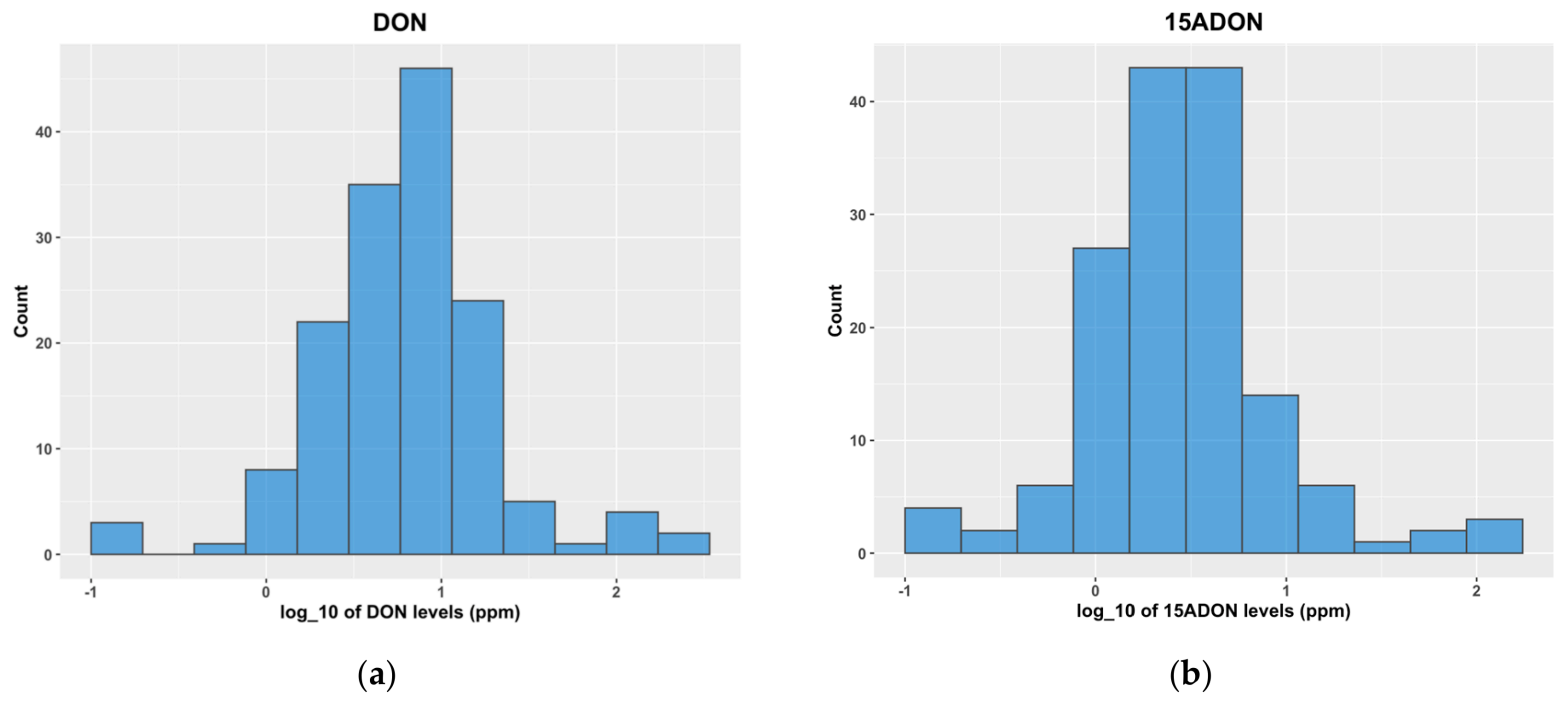
Distribution of least square means for the in vitro levels of DON and 15ADON for the tested isolates: (**a**) Histogram of the log10 of the least squares means of DON levels in ppm; (**b**) histogram of the log10 of the least squares means of 15ADON levels in ppm.

The results from three replicates were used to estimate least squares means, model-based averages across replicates that adjust for instances of missing data, for DON and 15ADON from each isolate. An analysis of variance showed that isolates produced significantly different levels of both DON and 15ADON (*p* < 10^−4^ in each case). The amount of DON produced, other than by the three DON non-producers, ranged from 0.7 ppm by isolate 19137 to 340 ppm produced by isolate 23356. The average amount of DON produced was 15 ppm and the median was 6.2 ppm (Figure 1a). Similarly, the amount of 15ADON produced, other than by the three 15ADON non-producers, ranged from 0.17 ppm by isolate 23365 to 174 ppm by isolate 23448 (Figure 1b). The average level of 15ADON was 7.4 ppm and the median was 2.8 ppm. Isolate 23344, the only isolate with the 3ADON *TRI* genotype, produced the largest amount of 3ADON (17 ppm averaged across three replicates).

### 2.3. Genome-Wide Associations

**Figure 2:**
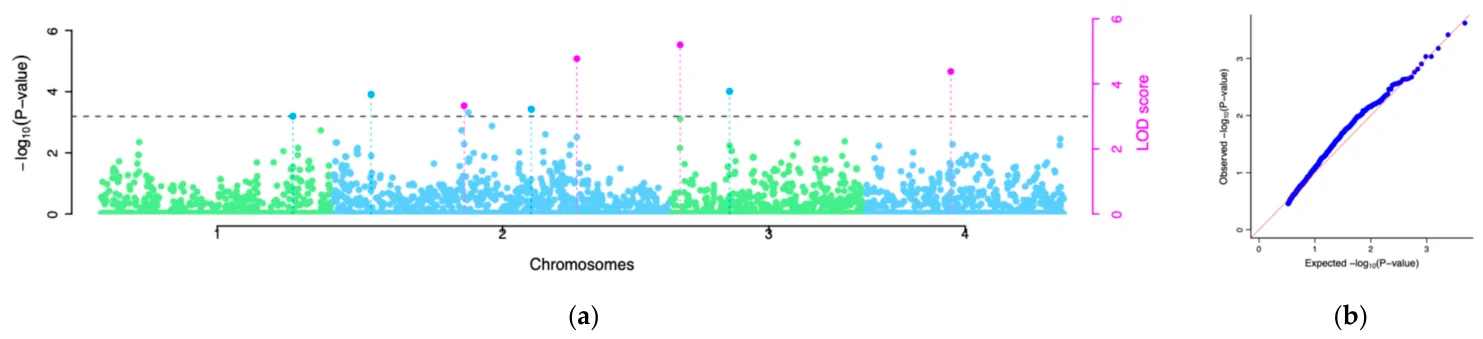
GWAS results for in vitro DON levels: (**a**) Manhattan plot displaying marker–trait associations across 151 isolates for in vitro DON levels. The *x*-axis displays the chromosome and position of each SNP tested, and the *y*-axis displays −log_10_(*p*-value) and LOD scores. SNPs with significant LOD scores have dotted vertical lines that drop down to the *x*-axis, and those significant in a single model have LOD scores plotted in bold blue, while those significant in two or more models have LOD scores plotted in pink. The remaining points (light green, light blue) represent the median of the intermediate −log_10_(*p*-values) from the models mrMLM, FASTmrMLM, FASTmrEMMA, and pKWmEB. The dashed horizontal line marks the LOD = 3 significance threshold; (**b**) Q-Q plot of observed over expected −log10 _10_ (*p*-values) for each SNP as a blue dot for in vitro DON levels among the isolates tested. The solid red line is for reference and indicates where observed values equal expected values.

**Figure 3:**
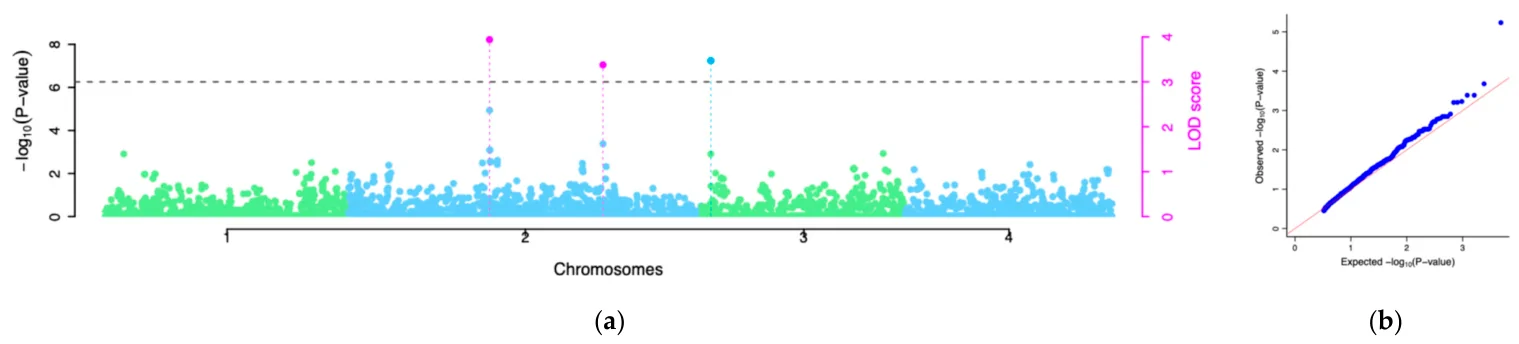
GWAS results for in vitro 15ADON levels: (**a**) Manhattan plot displaying marker–trait associations across 151 isolates for in vitro 15ADON levels. The *x*-axis displays the chromosome and position of each SNP tested, and the *y*-axis displays −log_10_(*p*-value) and LOD scores. SNPs with significant LOD scores have dotted vertical lines that drop down to the *x*-axis, and those significant in a single model have LOD scores plotted in bold blue, while those significant in two or more models have LOD scores plotted in pink. The remaining points (light green, light blue) represent the median of the intermediate −log_10_(*p*-values) from the models mrMLM, FASTmrMLM, FASTmrEMMA, and pKWmEB. The dashed horizontal line marks the LOD = 3 significance threshold; (**b**) Q-Q plot of observed over expected −log_10_ (*p*-values) for each SNP as a blue dot for in vitro 15ADON levels among the isolates tested. The solid red line is for reference and indicates where observed values equal expected values.

Multi-locus GWAS models implemented in the R package mrMLM [15] detected eight and three significant SNPs for DON and 15ADON, respectively (Figure 2a and Figure 3a; Table 1 and Table 2). Diagnostic quantile–quantile (Q-Q) plots verified that association *p*-values were not inflated due to population structure (Figure 2b and Figure 3b). Multiple QTL were detected by more than one model implemented in the package. For the trait DON concentration, four QTL were detected by two of the implemented models. Comparing across the GWAS models, ISIS EM-BLASSO detected five QTL, pKWmEB detected four, FASTmrMLM detected two and mrMLM detected a single QTL. QTL explained up to 10% of the phenotypic variation. Likewise, for 15ADON concentration, one QTL was detected by all five models implemented, one QTL was detected by two different models (pKWmEB and ISIS EM-BLASSO), and one QTL was detected only by ISIS EM-BLASSO. QTL explained up to 12% of the phenotypic variation for 15ADON levels. There is one common QTL for DON and 15ADON levels, QTL 6, on chromosome 2.

### 2.4. Candidate Genes

A list of candidate genes from the annotation of the PH-1 reference genome overlapping or close to each significant SNP was generated based on patterns of LD in our sample and in the NA1 population generally. For each QTL, we defined LD blocks flanking each side of the SNP with a minimum of 3 kb in length, and a greater length for QTL where our sample exhibited longer-range LD. Across all significant SNPs, the size of the LD blocks on each side of the SNPs ranged from 3 to 17 kb. The number of candidate genes identified within these LD blocks across the 10 distinct QTL ranged from two to sixteen, for a total of 50 distinct candidate genes (Table S2). Due to the proximity of QTL 7 and 8 on chromosome 3 (just over 3 kb apart), one candidate gene is shared by these two QTL. Nineteen of the candidate genes are annotated as hypothetical proteins, another 11 are annotated as unnamed protein products, but the remaining 20 candidate genes had more informative gene annotations. Additionally, gene ontology (GO) terms were available for 24 of the candidate genes, including a few of the hypothetical proteins and unnamed protein products (Table S2). This set included 12 genes with a GO cellular component term, 20 with a GO molecular function term, and 15 with a GO biological process term.

## 3. Discussion

An important goal of this study was to measure variation in trichothecene levels across a large sample of *F. graminearum* isolates grown under uniform in vitro conditions. The size of our sample and our controlled experimental design are strengths of this study and compare favorably with previous studies of population variability in trichothecene levels. The data were then used to identify QTL associated with this variation through GWAS implemented in multiple multi-locus models and to identify candidate genes. We previously identified thousands of SNPs evenly distributed across the genome for use in association testing and for characterization of the population structure of a larger *F. graminearum* sample [11]. A subset of more genetically homogeneous isolates, primarily from the NA1 population, were used in our GWAS analyses here. We identified 10 distinct QTL associated with mycotoxin levels and found candidate genes based on their proximity to these QTL. Our investigation of these results provides insights into the genetic basis of differences in trichothecene levels.

The levels of trichothecenes found on crops naturally infected by *F. graminearum* in the field are variable and are heavily influenced by environmental factors [9]. Even when *F. graminearum* is cultured in the laboratory on grain substrate, the levels of DON detected span a wide range and depend on substrate, temperature, moisture, and incubation time [16]. In the current study, all experimental replicates were conducted in the same laboratory space, and all isolates within each replicate were grown at the same time, so that the ambient environmental conditions such as temperature are reasonably uniform across all isolates. The DON levels from 15ADON strains had a mean of 15 ppm and median of 6.2 ppm, while the mean and median of 15ADON from the same strains was 7 and 3 ppm, respectively. The highest level of DON produced by any of our 15ADON isolates in the current study was 340 ppm and the highest level of 15ADON was 174 ppm. In comparison, three previous studies conducted by two different groups at North Dakota State University, that used a similar experimental design on fewer *F. graminearum* isolates grown on rice substrate, resulted in higher DON and 15ADON levels on average [17,18,19]. The mean and median amounts of DON detected from 15ADON *F. graminearum* isolates in each of these three studies are 166 and 84 ppm [17], 168 and 152 ppm [18], and 127 and 113 ppm [19], respectively. The average and median amounts of 15ADON produced from the same samples of these three studies are 35 and 8 ppm [17], 8 and 7.7 ppm [18], and 8.5 and 9 ppm [19], respectively. The highest levels of DON in the previous studies when including only isolates with a 15ADON genotype exceeded 400 ppm in only one study, with 782 ppm from [17], while the maximum 15ADON level exceeded 20 ppm only in that same study, with 322 ppm [17,18,19]. Seven of the 54 isolates with 15ADON genotypes from these three studies produced more DON than any of the isolates in our study [17,18,19].

Given the high sensitivity of DON levels to environmental factors, it is not unexpected that DON levels in rice cultures, even for the same set of isolates, could vary by laboratory. The variety of rice may matter: we used Ben’s Original parboiled rice, while the other studies do not report the type of rice. We used less rice, 20 g rather than the 30 g used in the other studies. We hydrated our rice in a fixed volume of water, rather than using much more and draining off the excess after 6 or 10 h, which could have led to different amounts of moisture available at the beginning of the incubation period. We inoculate the flasks with plugs of mung bean agar cultures, while one study inoculates with potato dextrose agar plugs and the other two inoculate with 2.5 × 10^6^ macroconidia. Only one study [18] gives a specific incubation temperature rather than indicating room or ambient temperature, so the incubation temperature and humidity conditions may have differed. We harvested the cultures after 21 days of incubation, while the other studies incubated for 28 or 30 days. These differences likely contribute to the differences in DON and 15ADON between other published studies and our results. Some variation in DON and 15ADON levels in different studies is also expected due to differences in the origin and genetic background of the isolates included. For our study, the relative values of the DON and 15ADON levels compared to other isolates in our sample are much more important than the absolute values, as the GWAS detects SNPs associated with relatively high or low trichothecene levels rather than requiring absolute toxin levels. Nonetheless, our GWAS results are specific to our experimental measurements, and will not necessarily match those from experiments in other laboratories. Similarly, our candidate QTL may also be specific to in vitro conditions as opposed to mycotoxin levels that result from greenhouse or field inoculations of wheat, since not only would those environmental conditions vary more, but the plant–fungus interactions on the wheat head could also influence DON levels differently across our strain sample. How applicable these candidate QTL will be to predicting mycotoxin levels on wheat heads with FHB will be determined only after comparing with a future GWAS study that analyzes DON levels detected from wheat heads infected with these same isolates.

Models implemented in the R-package mrMLM identified eight QTL associated with DON levels and three QTL associated with 15ADON levels. One QTL was common to both mycotoxins, making 10 unique QTL associated with variation in trichothecene levels in this study. Five of the ten QTL were detected by more than one model, including the QTL common to variation in both DON and 15ADON levels, increasing the likelihood that these QTL play a causative role in determining mycotoxin levels in our laboratory experiments.

In *F. graminearum*, genes in three *TRI* gene clusters code for and regulate the production of trichothecene mycotoxins [6], and nucleotide variation in these genes can alter the composition of trichothecenes produced [6]. None of the QTL identified in this study were located near any of the *TRI* clusters (Table S2). The QTL regions identified include several potentially interesting candidate genes, including several encoding transmembrane transporters that may be important for trichothecene export as well as fungal self-protection against trichothecenes by sequestration in vesicles and vacuoles [10,20,21].

Gene FGRAMPH1_01G13371, a major facilitator superfamily (MFS) transporter gene in the LD block of QTL 5 on chromosome 2, is significantly upregulated during FHB infection and could be important in DON levels and aggressiveness [22]. Though not identified as a QTL in this study, *TRI12* is also an MFS transporter associated with DON accumulation and self-protection from DON [10]. As additional evidence of the importance of these types of genes, 33 transporter genes, 15 of which are MFS genes, are upregulated when DON is applied exogenously to cultures [23]. Additionally, the deletion of the ATP-binding cassette transporter gene *Abc1* reduces DON accumulation and virulence on wheat, while deletion of *Abc6* also reduces virulence [21]. Genes FGRAMPH1_01G08381 near QTL 1, FGRAMPH1_01G12019 near QTL 3 and FGRAMPH1_01G12139 near QTL 4 are all predicted to be integral membrane components and transmembrane transporters. Following their synthesis in toxisomes, it is thought that vesicles with transporters accumulate and concentrate trichothecenes and move them to vacuoles for storage or the plasma membrane for exocytosis [9]. The enrichment of cellular transport genes in the set of 153 genes upregulated when cultures are exposed to a trichothecene biosynthesis intermediate provides support that transporters are involved in DON accumulation and self-protection [24]. The transporter genes among our candidates could have important roles in transporting trichothecenes for storage or for release out of the fungal cell, and variation in their self-protection capabilities could contribute to the variation in DON levels that we see.

Genes FGRAMPH1_01G08375, FGRAMPH1_01G08377, and FGRAMPH1_01G08379 in the LD block for QTL 1 and genes FGRAMPH1_01G14139 and FGRAMPH1_01G14141 near QTL 6 are predicted to be members of secondary metabolite clusters C61 and C18, respectively [25]. These clusters code for as yet unidentified secondary metabolites. Association of these genes with trichothecene levels suggests interconnections between the production and regulation of multiple secondary metabolites in *F. graminearum*. Transporters coded outside the *TRI* gene clusters have a known role in toxin efflux and accumulation [21]. We hypothesize that *F. graminearum* transmembrane transporters in other secondary metabolite synthesis clusters transport trichothecenes between organelles or for excretion and serve a role in self-protection against trichothecenes.

QTL 9 on chromosome 3 for DON levels has in its LD block the gene FGRAMPH1_01G18675 which encodes Swr1p, a helicase that is the catalytic subunit of the SWR1 complex involved in H2Z.A deposition [26]. Mutations in this gene lead to reduced DON levels [27]. Currently, we are unsure about the potential roles of genes in the LD blocks of QTL 2, 7, 8 and 10 in DON and 15ADON levels. Annotated genes near these QTL encode NADPH dehydrogenase 2 (FGRAMPH1_01G09217) near QTL 2, Heterokaryon incompatibility OR allele (FGRAMPH1_01G16145) near QTL 7, and l-amino-acid oxidase (FGRAMPH1_01G16149) near QTL 8. All other genes near QTL 2, 7, and 8, and all genes near QTL 10 code for hypothetical or unknown proteins (Table S2).

The candidate genes in this study were identified by their proximity to GBS-derived SNP markers associated with variation in DON and 15ADON levels in *F. graminearum.* Additionally, a well-known limitation of association analysis is the potential for false positive associations due to such factors as the confounding effect of genetic background [14]. Thus, the potential effects of the candidate genes on levels of DON remain to be functionally validated. Since the GBS sequence data does not include the full sequence of these genes and their flanking regions, a first step would be to generate this sequence across our sample, test for the trait associations of these variants and predict their functional effect on each gene. Subsequently, several strategies could be taken to functionally validate the involvement of these genes in trichothecene levels or to confirm the functional effect of substituting alternative alleles segregating at each candidate gene in our sample. The candidate gene expression could be measured via RNA-Seq or quantitative real-time polymerase chain reaction (qRT-PCR) under either trichothecene inducing conditions or after treatment with exogenous DON to determine if they are upregulated under these conditions. While none of our candidate genes were found to be upregulated by exogenous DON or the trichothecene intermediate trichodiene [23,24], the genetic background of the strains might impact the potential upregulation. Differential expression of candidate genes relative to housekeeping genes would provide further evidence that the genes are important for variation in trichothecene levels. Similar approaches have been used to identify genes important for infection in wheat and other hosts [28,29] as well as to understand the differential transcriptional profiles in 3ADON and 15ADON producing isolates [30]. Targeted gene disruption or allele replacement studies can also be used for gene function validation. In F*. graminearum*, functional characterization of genes has traditionally relied on targeted gene deletion by homologous recombination, or insertional mutagenesis, followed by complementation with a functional gene copy [24,31,32] and more recently on CRISPR-Cas9-mediated gene editing [33,34]. These approaches have successfully been used to validate the genes required for virulence, fertility, development and mycotoxin synthesis. A similar approach to replace one gene variant with another can be used to confirm the variant’s effect on variation in DON and 15ADON levels. The predicted transmembrane transporter genes FGRAMPH1_01G13371 (QTL 5), FGRAMPH1_01G08381 (QTL 1) FGRAMPH1_01G12019 (QTL 3), and FGRAMPH1_01G12139 (QTL 4) are likely our best candidates for functional validation. In fact, gene FGRAMPH1_01G13371 is upregulated significantly during FHB infection and could be important in DON synthesis and transport and aggressiveness [22]. We hypothesize that CRISPR-Cas9-mediated gene editing of these genes will lead to reduced DON levels in FGRAMPH1_01G13371 (QTL 5), FGRAMPH1_01G08381 (QTL 1), and FGRAMPH1_01G12019 (QTL 3), and reduced 15ADON levels in FGRAMPH1_01G12139 (QTL 4) in rice cultures inoculated with CRISPR-edited isolates. Their role in influencing trichothecene levels in wheat grains can be investigated by inoculating the plants with CRISPR-edited isolates and quantifying the trichothecenes in infected grains.

It is unlikely that we have identified all the loci segregating in our strains that influence DON or 15ADON levels even though our GBS data provided a set of dense genetic markers distributed throughout the genome and LD allows for the detection of causal variants that are not directly included in the GWAS analysis. Genetic recombination in *F. graminearum* is not distributed evenly across the genome but instead tends to cluster in hotspots [35]. Accordingly, the physical intervals associated with LD will vary, with strong associations between SNPs at greater distances in regions of low recombination and limited associations between nearby SNPs in regions of high recombination. Future work to completely sequence the genomes of the isolates in this study would help to more comprehensively map the QTL that influence trichothecene levels in this sample down to the quantitative trait nucleotide (QTN) level. Several previous studies that have measured DON levels from cultures grown on a solid rice substrate have included multiple strains from both the 3DON and 15ADON chemotypes, allowing for comparisons between these groups [17,18,19]. Our study has focused on strains from the *F. graminearum* NA1 population, resulting in all but one of our strains with genotypes consistent with the 15ADON chemotype. Work in the future to expand our sample to include more strains with different chemotypes would enable comparisons of the associated genetic variants and genes between the chemotype groups.

A previous study conducted GWAS for DON levels with *F. graminearum* samples, though with many relevant differences compared with our own study [36]. Of the 17 genes associated with DON levels from this previous study, none were found significantly associated in our own study [36]. The difference in results could be due to several factors. Most importantly, both the specific trait, DON from ground grain of spray-inoculated wheat plots detected by a commercial immunoassay kit, and the sample, 119 isolates from 13 German field populations, of the previous study differed from our work reported here [36]. The potential for significant genotype by environmental interactions for DON levels means that the relative levels among a sample of isolates for DON in a field plot could differ significantly from the relative levels obtained in the in vitro assay that we use. Also, the isolates we used were collected in the U.S. and come from a population that is presumably different from and evolving independently of the German population sampled in the other study [36]. Genetic variation in different sets of genes may underlie the phenotypic variation observed in the distinct populations. Additional experimental differences may also contribute to the differences in results. After dropping low frequency SNPs in both studies, our study includes just short of 5000 in the GWAS, less than one sixth of the more than 30,000 SNPs in the other study’s GWAS [36]. Even though the lower extent of linkage disequilibrium in the other study (SNPs ~ 1 kb away show minimal correlation levels [36]) corresponds to a higher SNP density required for the same genome coverage, our study appears to have a lower genome coverage and as a result may miss variants in the genome that contribute to variation in DON levels. This difference in genome coverage between the two studies could also contribute to their lack of overlap between associated genes.

By implementing multiple GWAS models, we identified several robust QTL and candidate genes that are associated with mycotoxin levels and that may contribute directly to them. These traits are important to the fitness of *F. graminearum* isolates and the safety of the food supply. Functional characterization of these genes will expand our understanding of molecular mechanisms driving mycotoxin synthesis and regulation.

## 4. Materials and Methods

### 4.1. Fungal Isolates

A total of 151 isolates of *Fusarium graminearum* collected by other researchers were used in this study [18,19,37,38,39,40,41] (Table S1). The inference of *TRI* loci genotype and population membership for each isolate have been previously described [11]. All isolates have been previously assigned to individual populations when their inferred ancestry coefficient corresponding to that population was 0.9 or greater [11]. These isolates came from the North America 1 (NA1) population, except for four isolates not assigned to a population because their inferred ancestry coefficient for each defined population was <0.9. These isolates were collected by other researchers between 1999 and 2013 primarily from Minnesota, Montana, North Dakota, and New York, with a single isolate coming from each of Virginia, Louisiana, and Michigan.

### 4.2. Genotyping

Isolates used here are a subset of the isolates from our 2024 population genomics study [11]. Isolates were sequenced with genotyping by sequencing (GBS) [11]. Polymorphisms were extracted using GATK 4.1.8.1 [42]. Missing genotype calls for all but sites with extreme levels of missing data were imputed using data from most of the isolates from our larger study with Beagle 5.1 [43]. SNPs were included in the imputation process if they had genotype calls in >25% of a set of 555 isolates. These 555 isolates come from the original 570 isolates sequenced in that study minus the six with the fewest genotype calls and an additional nine that differed from the PH-1 isolate at >10% of the identified SNPs. A total of 63,102 SNPs were used in the imputation process. The subsets of sites that were polymorphic for the set of isolates used in the mycotoxin experiment were obtained and filtered to exclude sites with minor allele frequencies less than 0.05. A total of 4836 SNPs were used in the association mapping of mycotoxin levels.

### 4.3. Measurement of In Vitro DON Levels

The quantity of DON, 3ADON, 15ADON and NIV mycotoxins were measured from in vitro cultures of *F. graminearum* isolates grown on a solid rice substrate [17,18,19] in three independent experiments, or experimental replicates. These experiments were set up with a randomized complete block design, and in their analysis isolate was treated as a fixed effect and replicate was treated as a random effect. The results of DON and 15ADON levels from each isolate from these three replicates were used to estimate least squares means to adjust for rare instances of missing data, using the R package lsmeans [44]. For each experimental replicate, 20 g of parboiled rice (Ben’s Original^TM^ Parboiled Long Grain White Rice) was soaked overnight in 25 mL water in a 250 mL conical flask two nights before inoculation. A day before inoculation, flasks with water-soaked rice were covered with a double-layered coffee filter that was secured around the neck with a rubber band. Flasks were autoclaved twice (20 min liquid cycle). Flasks were cooled to room temperature (21–24 °C) between the autoclave cycles. Inoculation was performed by taking 3 plugs (5 mm in diameter) from 4-day old *F. graminearum* cultures growing on mung bean agar plates. To allow for proper mixing of conidia with rice, conical flasks were shaken every other day for up to 1 week after inoculation. Flasks with rice cultures were incubated in the dark at the laboratory’s ambient room temperature for 21 days. At 21 days, rice cultures were transferred to a 50 mL centrifuge tube and frozen at −80 °C. Rice cultures were freeze-dried at −40 °C (Thermo Savant, ModulyoD benchtop dryer, Waltham, MA, USA) for four days followed by grinding to a fine powder with commercial blade coffee grinders.

Powdered rice cultures were used for mycotoxin analysis with gas chromatography–mass spectrometry (GC-MS). Analysis for DON, 15ADON, 3ADON, and NIV was conducted at the mycotoxin testing lab at Virginia Tech. A one-gram subsample of each lyophilized, homogenized rice sample was analyzed for the B trichothecenes DON, 3ADON, 15ADON and NIV [45]. Sample extraction and clean-up followed the methods of Tacke and Casper [46] and Mirocha et al. [47] with some modification. Samples were extracted in 10 mL of acetonitrile: water (84:16, *v*/*v*) and then derivatized for analysis by GC-MS [46]. All samples were analyzed on an Agilent 7890B/5977B gas chromatograph-mass spectrometer (Santa Clara, CA, USA) operating in selected ion monitoring (SIM) mode [45]. Extracts from non-inoculated rice cultures were used as the background matrix in the preparation of calibration standards and analyzed concurrently with experimental samples.

### 4.4. Genome-Wide Association Analysis

Genome-wide association analyses were conducted using the R package mrMLM 5.0.1 [15]. Five mrMLM 5.0.1 models, namely mrMLM, FASTmrMLM (FAST mrMLM multi-locus mixed linear model), FASTmrEMMA (fast multi-locus random-SNP-effect EMMA), pKWmEB (integrates Kruskal–Wallis test with empirical Bayes), and ISIS EM BLASSO (iterative modified-Sure Independence Screening EM-Bayesian Lasso) were used. Models from the mrMLM R package perform multi-locus genome-wide association analysis, where the effects of all markers are estimated simultaneously. They perform two steps. In the first step, each SNP is tested individually for association, treating marker effects as random, and a relaxed Bonferroni threshold is applied to select potentially associated markers. In the second step, a multi-locus mixed linear model estimates the effects of these selected markers using an empirical Bayes approach implemented via the expectation–maximization (EM) algorithm. Likelihood ratio tests were performed, and markers with LOD scores greater than or equal to 3 were considered significant. The optimal number of principal components to use for accounting for population structure was decided based on forward model selection results in GAPIT version 3 using the Bayesian information criterion and diagnostic quantile–quantile (Q-Q) plots [48]. For both traits used in GWAS here, the optimal number of principal components identified was zero, consistent with nearly all the isolates in our sample coming from the same genetic population.

### 4.5. Candidate Gene Analysis

The linkage disequilibrium (LD) block for each significant SNP was defined by starting with a minimum block size of 3 kb on each side of the SNP. This distance corresponds to the average LD decay patterns for the NA1 population, from which the sample in the GWAS analysis was drawn. Subsequently, we manually inspected the LD decay pattern beyond these minimum blocks on both sides of the SNP. The squared correlation coefficient (*r*^2^) between the significant SNP and a few SNPs closest to it on the left and right were used to determine whether to expand the left and/or right LD block size when strong LD was apparent beyond the minimum block. Total block size is the sum of left and right blocks. Genes falling in the LD block of the significant SNPs were obtained using a custom python script. The curated phenotypes and gene ontology terms for the candidate genes were obtained from FungiDB [49].

## Figures and Tables

**Table 1:** List of SNPs significantly associated with in vitro DON levels.

QTL No.	Chr	Position	Method	QTL Effect	LOD Score	−log_10_(*p*)	*r* ^2^	MAF
1	1	11,260,778	ISIS EM-BLASSO	−0.0001	3.01	3.70	0.00	0.18
2	2	520,988	FASTmrMLM	−0.13	3.67	4.41	3.90	0.19
3	2	4,175,663	FASTmrMLM	0.16	3.11	3.82	4.37	0.15
			ISIS EM-BLASSO	0.07	3.54	4.27	0.86	
5	2	5,687,139	pKWmEB	0.13	3.22	3.93	7.62	0.20
6	2	6,526,109	ISIS EM-BLASSO	0.13	5.83	6.66	3.92	0.21
			pKWmEB	0.13	3.72	4.46	10.15	
8	3	102,121	ISIS EM-BLASSO	−0.19	5.49	6.31	5.44	0.12
			pKWmEB	−0.18	4.90	5.70	9.89	
9	3	3,285,338	mrMLM	0.20	3.77	4.51	5.87	0.12
10	4	4,261,526	ISIS EM-BLASSO	0.12	5.71	6.54	3.97	0.25
			pKWmEB	0.10	3.05	3.74	6.53	

Chr, chromosome; QTL effect, estimated effect of the allele on the trait measure; LOD score, logarithm of odds; *r*^2^, percent of phenotypic variance explained by each QTL; MAF, minor allele frequency of the SNP.

**Table 2:** List of SNPs significantly associated with in vitro 15ADON levels.

QTL No.	Chr	Position	Method	QTL Effect	LOD Score	−log_10_(*p*)	*r* ^2^	MAF
4	2	4,313,942	mrMLM	−0.30	5.00	5.79	12.77	0.11
			FASTmrMLM	−0.25	3.79	4.53	8.98	
			FASTmrEMMA	−0.44	3.94	4.69	7.00	
			pKWmEB	−0.23	3.66	4.40	11.46	
			ISIS EM-BLASSO	−0.22	4.25	5.01	7.38	
6	2	6,526,109	pKWmEB	0.02	3.13	3.83	6.52	0.21
			ISIS EM-BLASSO	0.12	3.63	4.36	4.00	
7	3	98,785	ISIS EM-BLASSO	−0.15	3.47	4.20	2.78	0.09

Chr, chromosome; QTL effect, estimated effect of the allele on the trait measure; LOD score, logarithm of odds; *r*^2^, percent of phenotypic variance explained by each QTL; MAF, minor allele frequency of the SNP.

## Data Availability

Information on all strains used in the experiments have been included in a Supplemental Table, including the SRA accession numbers for the publicly available DNA sequence data used in this study. Additional supporting data on isolate genotypes and phenotypes, and code written for the analyses described here are made available on GitHub: https://github.com/Toomajian-laboratory/files_mycotoxin/ (accessed on 1 December 2025).

## Supplementary Materials

The following supporting information can be downloaded at https://www.mdpi.com/article/10.3390/toxins18080322/s1. Table S1: Information about isolates used in this study; Table S2: List of candidate genes flanking significant SNPs.

## Abbreviations

The following abbreviations are used in this manuscript:

3ADON	3-acetyl-deoxynivalenol
15ADON	15-acetyl-deoxynivalenol
DON	deoxynivalenol
EM	expectation–maximization
FHB	Fusarium head blight
GBS	genotyping by sequencing
GC-MS	gas chromatography–mass spectrometry
GWAS	genome-wide association study
LD	linkage disequilibrium
LOD	logarithm of odds
LOQ	limit of quantitation
NA1	North America 1
NIV	nivalenol
ppm	parts per million
Q-Q	quantile–quantile
qRT-PCR	quantitative real-time polymerase chain reaction
QTL	quantitative trait locus
QTN	quantitative trait nucleotide
SIM	selected ion monitoring
SNP	single nucleotide polymorphism
